# Workplace Loneliness Experience Among Older Professionals (Aged ≥50 Years) in the Context of Digitalization: Protocol for a Scoping Review

**DOI:** 10.2196/81843

**Published:** 2025-12-25

**Authors:** Lavinia Iuliana Țânculescu-Popa, Maria Piedade Brandão, Şeyhmus Aksoy, Rogério Pessoto Hirata, Ayşegül Ilgaz, Cristina Maria Tofan

**Affiliations:** 1 Faculty of Psychology and Educational Sciences Hyperion University Bucharest Romania; 2 Faculty of Communication and Public Relations National School of Political Science and Public Administration Bucharest, București Romania; 3 Health School of University of Aveiro (ESSUA) Aveiro Portugal; 4 RISE-Health, Health School of University of Aveiro Aveiro Portugal; 5 Department of Psychology, Faculty of Science and Letters Agri Ibrahim Cecen University Ağrı Turkey; 6 ExerciseTech Research Group Department of Health Science and Technology Aalborg University Aalborg Denmark; 7 Department of Public Health Nursing Faculty of Nursing Akdeniz University Antalya Turkey; 8 Psychology and Educational Sciences Department Gheorghe Zane Institute for Economic and Social Research Romanian Academy - Iasi Branch Iasi Romania; 9 Department of Sociology, Social Work and Human Resources Faculty of Philosophy and Social-Political Sciences ”Alexandru Ioan Cuza” University Iasi Romania

**Keywords:** workplace loneliness, digital tools, digital technology, older workers, scoping review

## Abstract

**Background:**

Workplace loneliness—defined as the perceived absence of meaningful social relationships at work—can have a negative impact on the well-being, engagement, and productivity of employees. Older professionals (aged ≥50 years) may be particularly vulnerable to workplace loneliness in the context of accelerated digitalization, which may create obstacles to inclusion, communication, and collaboration. Despite growing interest in this phenomenon, no comprehensive synthesis has yet examined how digital tools and transformations affect loneliness among older workers or what interventions have been implemented to address it.

**Objective:**

This scoping review aims to systematically map the existing literature on workplace loneliness among workers aged 50 years or older, with a particular focus on how digitalization influences these experiences. The review will also identify digital tools associated with loneliness and explore organizational interventions to reduce loneliness among this demographic.

**Methods:**

This scoping review will follow the methodological framework by Arksey and O’Malley and the PRISMA-ScR (Preferred Reporting Items for Systematic Reviews and Meta-Analyses extension for Scoping Reviews) reporting guidelines. A comprehensive search will be conducted in multiple databases (MEDLINE, Scopus, Web of Science, and APA PsycINFO) and gray literature. Eligible studies will (1) include workers aged 50 years or older, (2) address workplace loneliness or related constructs in professional contexts, and (3) be situated in digitalized work environments (eg, remote or hybrid work, digital tools, and information and communications technology systems). Study selection, data extraction, and quality assessment will be conducted independently by at least 2 reviewers. Data will be charted using a predefined template covering study characteristics, theoretical frameworks, the digital context, loneliness measures, and intervention strategies and synthesized narratively and thematically.

**Results:**

As this is a scoping review protocol, results are not yet available. A preliminary search conducted in June to July 2025 across MEDLINE, Cochrane Library, and Web of Science yielded 450, 1, and 47 potentially relevant records, respectively. No systematic or scoping reviews were identified on workplace loneliness among older workers in digitalized contexts. One review addressed video calls for nonworking older adults, reporting very low-certainty evidence. The planned review will apply the PRISMA-ScR guidelines to synthesize evidence on digitalization’s role in, technologies associated with, and organizational interventions to mitigate workplace loneliness for professionals aged 50 years or older.

**Conclusions:**

This scoping review will systematically examine how digitalization shapes workplace loneliness among professionals aged 50 years or older and identify organizational interventions that address it. The synthesis will refine conceptual understanding, highlight critical evidence gaps, and inform the development of socially supportive digital work environments for aging workforces.

**Trial Registration:**

Open Science Framework 6p4ak; https://osf.io/6p4ak

**International Registered Report Identifier (IRRID):**

DERR1-10.2196/81843

## Introduction

### Background

As workplaces become increasingly reliant on digital communication technologies, older workers (aged ≥50 years) may experience challenges in adapting, which can influence their sense of belonging and overall workplace experience [1]. This issue is significant given the crucial role of older professionals in mentoring and knowledge transfer. Many expect to remain in the workforce for 15 years or more; therefore, ensuring their inclusion and connectedness is vital [2-4].

The aging workforce represents a global demographic shift. United Nations projections indicate that the number of older working-age individuals (aged 55-64 years) will rise by nearly 49% between 2021 and 2050 and by 68% by 2100 [5]. As people live longer and work later, organizations face new challenges in sustaining well-being and productivity. One key concern is workplace loneliness, which can affect older employees’ satisfaction, engagement, and performance [6].

Workplace loneliness refers to the perceived absence of meaningful social relationships at work, encompassing both emotional and social isolation [7,8]. It is associated with reduced job satisfaction, lower engagement, and diminished performance [9,10]. Loneliness is not merely physical separation but a subjective sense of disconnection stemming from unmet social needs [11-13]. While loneliness has often been studied as an individual experience, organizational factors such as leadership, culture, and communication systems play an equally important role. The digitalization of work—through online platforms, automation, and remote communication—has transformed how employees interact [14]. While these innovations offer several benefits, including increased flexibility and efficiency, they can also contribute to workplace stress, strain, and negative impacts on social connection, particularly among older workers who may struggle to adapt to rapidly changing technological environments [15].

Sociocognitive models argue that loneliness emerges when there is a mismatch between expected and actual social interactions, which are shaped by self-perception, cognitive biases, and attributional tendencies [16]. Life span developmental theories emphasize that older adults may become more selective in their social investments, focusing on emotionally meaningful interactions over broader social networks [17]. In organizational theory, structural factors such as hierarchical distance, leadership style, and digital communication norms can buffer or intensify loneliness [18]. Together, these frameworks highlight the interplay between individual psychology and workplace structures in shaping later-career experiences.

Older employees (aged ≥50 years) often face distinctive challenges in digital environments. Low digital literacy can create feelings of exclusion and limit communication, leading to weaker professional ties [19]. The expansion of remote and hybrid work since the COVID-19 pandemic has further intensified reliance on digital interaction, potentially increasing isolation among older workers [20]. While workplace loneliness is often viewed through a negative lens, recent scholarship has called for a distinction between distressing loneliness and restorative solitude. Solitude, when chosen rather than imposed, can foster creativity, emotional regulation, and a sense of autonomy [21,22]. For older professionals, the ability to step back from constant digital interaction may offer moments of reflection and recharge, contributing to their well-being and professional insights [23]. Therefore, organizations should be cautious not to pathologize every experience of being alone, recognizing that solitude can, in some cases, act as a psychological resource [24].

The effects of workplace loneliness are significantly shaped by how the concept is operationalized. When defined narrowly as a painful emotional experience due to unwanted isolation, loneliness is consistently linked to negative outcomes, including stress, depression, and disengagement [25]. However, broader definitions that include aspects of solitude or intentional aloneness reveal a more ambivalent picture. For instance, individuals high in introversion may prefer limited social interaction yet remain satisfied and productive [26]. Understanding these definitional boundaries is crucial for interpreting empirical findings and designing nuanced interventions that distinguish between detrimental loneliness and adaptive solitude.

Recent scholarship emphasizes that workplace loneliness is qualitatively distinct from general loneliness as it emerges from the specific relational and structural dynamics of professional environments [27]. This distinction is important as context-specific drivers such as organizational culture, leadership behavior, and the integration of digital tools can shape loneliness in ways not captured by general measures [28,29]. In older workers, reduced opportunities for informal interaction in digitalized workplaces may impact both psychological well-being and job engagement. Furthermore, social isolation in professional contexts has been associated with higher turnover rates and diminished organizational commitment, which can disrupt workforce stability [30].

Although digital technologies can foster connectivity, their benefits are unevenly distributed. Employees with lower digital literacy risk exclusion and marginalization [31]. Conversely, inclusive communication strategies, empathetic leadership, and targeted digital skill training can help counteract these effects [32,33]. Consequently, the literature underscores the need to design digital inclusive workplaces that balance efficiency with human connection, ensuring that older workers remain integrated, valued, and engaged in an increasingly digital working world [34].

### Goal of the Research

This scoping review aims to address the research gap in understanding workplace loneliness among older workers (aged ≥50 years), with a particular focus on the influence of digitalization. It will synthesize state-of-the-art evidence to explore how digitalization shapes these experiences—both positively and negatively—and identify effective, documented interventions to reduce workplace loneliness. By charting the existing literature and examining organizational strategies, the review will offer practical insights for supporting the aging workforce and enhancing their work experience in increasingly digital environments.

## Methods

### Guidance and Framework

To ensure methodological rigor and transparency in the reporting of this scoping review, we will follow the PRISMA-ScR (Preferred Reporting Items for Systematic Reviews and Meta-Analyses extension for Scoping Reviews) guidelines [35]. This protocol closely follows the structure and reporting logic applied in recent related work within the DIGI-net network, particularly the methodological approach described by Spijker et al [36]. Their work provided a valuable reference point for consistency and transparency in the scoping review design. Methodologically, this scoping review will be conducted following the guidance of Arksey and O’Malley [37] and guided by the recommendations of Levac et al [38], consisting of the following stages: (1) defining the research question, (2) identifying pertinent studies, (3) selecting studies based on inclusion criteria, (4) extracting and synthesizing data, and (5) summarizing and presenting the results. The checklist for this protocol is provided in Multimedia Appendix 1 [39].

### Protocol and Registration

This protocol was developed before conducting the full electronic literature search, which included identifying existing scoping reviews and pilot-testing search terms (see details below). This study was registered with the Open Science Framework [40].

### Stage 1: Identifying the Research Questions

We posed the following overarching research question: how does workplace digitalization influence experiences of loneliness among older workers (aged ≥50 years)? To address this, we also formulated three specific questions: (1) what is known from the existing literature about workplace loneliness in older workers in the context of digitalization? (2) What digital tools are used, and are they directly linked to or mediate experiences of loneliness? (3) What interventions have proven effective in reducing workplace loneliness among older professionals, and how have organizations implemented them?

On the basis of these research questions, we defined the population, concept, and context (PCC) criteria to clarify the focus of this scoping review and guide an effective search strategy [38] (Textbox 1 [41-46]). Regarding the population, our review will focus on studies involving older workers aged 50 years or older.

**Definitions of the inclusion criteria regarding population, concept, and context for this scoping review.**

**Population: older workers**

1. In this review, older workers are defined as individuals aged 50 years or older who were actively engaged in paid employment at the time the study was conducted. This threshold aligns with previous research identifying 50 years or older as a meaningful demarcation of later-stage employment due to physiological, career, and social transitions associated with aging [41].
2. Studies will be eligible if they focus entirely on participants aged 50 years or older or if they include this age group in a way that allows for separate analysis of findings by age. When age is reported in ranges, the lower bound of the relevant category must include 50 years (eg, 50-59 years). The decision to adopt this threshold is grounded in evidence of the growing representation of this demographic in the labor market and their diverse employment trajectories and motivations [42].
3. If age is reported as a continuous variable, inclusion will apply only when the reported descriptive statistics (eg, range, mean, or median) indicate that the sample includes participants aged 50 years or older. Studies focusing exclusively on younger adults (<50 years) or on individuals who are retired or not engaged in paid professional work will be excluded.

**Concept: workplace loneliness**

1. Eligible studies will examine workplace loneliness, professional isolation, solitude, or emotional disconnection as they occur in relation to professional roles and structures.
2. Workplace loneliness is understood as a subjective experience of social isolation or lack of meaningful connection with colleagues, distinct from but related to social or personal loneliness [43]. It has been linked to reduced engagement, decreased performance, and health risks in organizational settings.
3. Studies focusing solely on non–work-related loneliness (eg, familial or social) will be excluded, as will those examining workplace factors without specific reference to loneliness or isolation.

**Context: digitalized work environments**

1. Digitalized work environments refer to professional settings in which work tasks, processes, and communication are mediated or transformed through the use of digital technologies. This includes but is not limited to remote and hybrid work arrangements, the use of digital communication platforms (eg, Zoom and Microsoft Teams), and the integration of information and communications technology in everyday operations [44].
2. Digitalization encompasses the adoption of digital workplaces, automation, and broader digital transformation initiatives, which can reshape organizational structures [45] and influence the nature of professional relationships [46].
3. For the purposes of this review, digitalized work environments are defined as any setting in which digital tools or systems play a central role in enabling, organizing, or delivering work. Studies that do not address digitalization or that examine purely traditional, nondigital workplaces will be excluded.

### Stage 2: Identifying Relevant Studies

This stage will involve a comprehensive and systematic search to identify relevant studies that meet the eligibility criteria defined in stage 1. The search strategy will be developed in accordance with the Joanna Briggs Institute Manual for Evidence Synthesis [47] and guided by the PCC framework, ensuring both breadth and focus in capturing appropriate evidence related to the review objectives. Our initial search on the main databases (ie, MEDLINE, Cochrane, ProQuest [including Dissertations and Theses Global], Web of Science, Scopus, APA PsycINFO, and ERIH PLUS) yielded no ongoing or completed systematic or scoping reviews on this topic.

To enhance comprehensiveness, the final search will also include additional databases, such as Embase, CINAHL, IEEE Xplore, and the ACM Digital Library, which index studies relevant to health, technology, and workplace digitalization. Further searches may be conducted in relevant gray literature repositories and institutional sources if deemed necessary during the screening process.

The search terms to be used are as follows: (“workplace loneliness” OR “occupational loneliness” OR “job-related loneliness” OR “professional isolation” OR “workplace isolation” OR “social isolation at work” OR “loneliness at work” OR “employee loneliness” OR “staff loneliness” OR “colleague isolation” OR solitude OR “workplace solitude” OR “professional solitude”) AND (“older professional*” OR “older employee*” OR “older worker*” OR “senior professional*” OR “senior employee*” OR “senior worker*” OR “aging professional*” OR “ageing professional*” OR “aged worker*” OR “older adult*” OR “older staff” OR “elderly worker*” OR “elderly employee*” OR “older personnel”) AND (“digitalization” OR “digitalisation” OR “digital transformation” OR “digital technology” OR “information and communication technology” OR ICT OR “technology adoption” OR “digital tools” OR “online communication” OR “virtual communication” OR “digital” OR app* OR web OR internet OR tech* OR “social media” OR chat OR online* OR cyber OR virtual OR “computerized” OR “computerised” OR electronic)

In our search query, we introduced symbols specific to Boolean operators such as OR to encompass a broad range of related loneliness and digital technology concepts and terms referring to older workers. We also used the AND Boolean operator to overlap and identify studies that contain all the main concepts. Finally, in constructing our search strategy, we used the asterisk (*) symbol as an end-of-root-word truncation mark.

A preliminary search conducted in June 2025 and July 2025 in PubMed, Cochrane, and Web of Science Core Collection yielded 450, 1, and 47 results, respectively. Of all the studies, only 1 review included older people and digital technology use. A systematic review assessed the effectiveness of video calls in reducing loneliness and social isolation among adults aged ≥65 years [48]. However, this review did not focus on workers or workplace settings; instead, the included studies were all conducted in nursing homes with nonworking older adults. Despite evaluating digital technology use, particularly video calls via internet-enabled devices such as smartphones, tablets, and laptops, it found very low-certainty evidence that these interventions reduce loneliness, social isolation, or depression. Other studies identified focus on technology use as an interventional tool and are not specific to any occupational setting but rather to the general population [49,50]. This search strategy will be adapted for other databases (eg, Scopus, APA PsycINFO, and Web of Science [all collections]) using appropriate syntax and indexing systems. The final version of the search strategy will be documented in accordance with PRISMA-ScR and Joanna Briggs Institute reporting standards. Our search will be limited to studies published in English.

### Stage 3: Study Selection and Eligibility Criteria

After removing duplicate references in stage 2 using a reference management tool, the remaining citations will be imported into Covidence (Veritas Health Innovation) for systematic screening. The selection procedure will take place in 2 sequential stages. First, titles and abstracts will be screened. Second, the full texts of potentially eligible studies will be reviewed. Each record will be independently assessed by 2 reviewers at both stages in accordance with the predefined eligibility criteria. Studies that clearly meet these criteria, as well as those for which relevance is uncertain, will advance to the full-text stage.

Considering the expected number of search results, the screening workload will be distributed among all coauthors. Covidence will facilitate the allocation of records, the identification of conflicts, and the documentation of decisions. Any conflicts flagged by the software will be resolved through the involvement of a third reviewer. The research team will meet regularly online to address methodological questions and ensure consistent application of the inclusion and exclusion criteria throughout the process.

The overall selection workflow will be presented in a PRISMA-ScR flow diagram, which will also include the reasons for excluding studies at the full-text stage. Eligibility criteria are organized according to the PCC framework and complemented with additional parameters such as source type, language, time frame, work status, and outcomes of interest, as shown in Textbox 2.

**Eligibility criteria for the scoping review.**

**Inclusion criteria**

1. Population: professionals aged 50 years or older or studies with age-disaggregated data allowing for analysis of this group
2. Concept: workplace loneliness, professional isolation, solitude, or emotional disconnection in professional settings
3. Context: digitalized work environments (eg, remote or hybrid work, digital communication platforms, automation, and digital transformation)
4. Setting: professional or organizational contexts involving paid employment
5. Study type: original studies with any design or data type (quantitative, qualitative, or mixed methods), including dissertations and organizational reports with empirical data
6. Publication status: peer-reviewed journal articles, conference proceedings, dissertations, and reputable gray literature (eg, reports from reputable organizations)
7. Publication language: English
8. Time frame: published from 2000 onward
9. Full-text availability: full text accessible for review

**Exclusion criteria**

1. Population: studies focusing exclusively on younger workers (aged ≤49 years) or on retirees not engaged in professional work
2. Concept: general or personal loneliness unrelated to the workplace or studies on workplace factors without specific reference to loneliness or social disconnection
3. Context: nondigitalized work environments or settings without reference to technology-mediated changes in the workplace
4. Setting: informal, nonprofessional, or volunteer work contexts
5. Study type: protocols, narrative reviews, systematic reviews, opinion pieces, editorials, or nonempirical publications
6. Publication status: unpublished studies lacking peer review and low-quality gray literature without empirical data
7. Publication language: languages other than English
8. Time frame: published before 2000
9. Full-text availability: full text not accessible for review

### Stage 4: Charting the Data

The protocol authors developed and refined the initial data charting form. Levac et al [38] state that data extraction is an iterative process. Thus, we may revise the data charting form over time as we gain a better understanding of the data related to the research questions. Specifically, we may include extra items that are relevant for answering the research questions in the form. It was recommended that 2 researchers conduct a pilot test on 7 articles to assess the data extraction form [51]. Before implementation, the research team will review and discuss the charting form to check that it is complete and comprehensive. After piloting and refining the form, data will be extracted independently by 2 reviewers using a Microsoft Excel spreadsheet. Disagreements or inconsistencies in data extraction will be managed through the involvement of a third reviewer from the research team.

Data extracted from each included study will cover key bibliographic information (eg, author, year, and country or region); study characteristics (eg, design, population, sample features, and theoretical framework); and findings relevant to the review objectives, including definitions and measures of loneliness, digitalization context, key results, and intervention details when applicable. A comprehensive list of data items and their descriptions is provided in item 11 of the PRISMA-ScR checklist (Multimedia Appendix 1).

### Stage 5: Collating, Summarizing, and Reporting the Results

Following the guidance proposed by Levac et al [38], the fifth stage of this review will involve three interconnected activities: (1) synthesizing the evidence through both descriptive numerical summaries and qualitative thematic analysis; (2) examining the extracted data to identify results that address the primary research question, which will be presented in a narrative format; and (3) interpreting the findings in light of the supplementary research questions, with consideration of their practical relevance and potential policy implications.

Alongside the narrative synthesis, structured tables will be used to present an organized summary of the main results. The reporting of findings will follow the PRISMA-ScR guidelines [35] to ensure methodological transparency and consistency.

### Quality Assessment

According to Peters et al [52], the screening and inclusion procedure of many scoping reviews involves a broad range of sources. They also point out that formal risk-of-bias assessments, statistical pooling (meta-analysis), and evidence quality evaluations are not part of scoping reviews [52]. This scoping review will not formally evaluate the methodological quality or risk of bias of the included studies because the goal of this research is to map the body of literature on workplace loneliness among employees aged 50 years or older in a systematic manner, with a particular focus on how digitalization affects these experiences.

## Results

As this is a scoping review protocol, no results are available at this stage. The planned review will follow the PRISMA-ScR reporting guidelines to systematically map and synthesize evidence on workplace loneliness among professionals aged 50 years or older in digitalized work contexts. We anticipate identifying a wide range of empirical and conceptual studies exploring how digital tools, communication practices, and organizational changes influence older professionals’ social experiences at work.

The results of our inquiry will be disseminated through a scoping review, with the selection process illustrated in a PRISMA-ScR flow diagram. An anticipated version of the PRISMA-ScR flowchart has been included in Multimedia Appendix 2 to depict the planned study selection process. Numerical data will be added upon completion of the review. Extracted data will be presented in structured tables summarizing study characteristics and key variables (eg, digital context, age, and sector) and expanded upon in a thematic narrative synthesis. This synthesis will address the primary research question (how does workplace digitalization influence experiences of loneliness among older workers?), as well as the following subquestions: (1) what is known from the literature about workplace loneliness in older professionals in the context of digitalization? (2) Which digital tools are associated with or mediate experiences of loneliness? (3) What organizational interventions have been effective in reducing workplace loneliness among older professionals, and how have they been implemented? Where possible, subgroup trends by gender, sector, or region will also be highlighted. The review aims to provide a comprehensive map of current knowledge and identify priority areas for future research.

## Discussion

### Existing Evidence and Research Gap

The existing literature suggests that, while the effects of digitalization on older workers are increasingly documented, workplace loneliness remains a relatively neglected focus of investigation. Previous studies have tended to examine aging, digital skills, or occupational well-being in broader terms, but few address the specific emotional and relational consequences of digital transitions—such as reduced informal contact, social disconnection, or perceived isolation—among professionals aged 50 years or older [27-29]. The evidence base is fragmented across disciplines, including public health, education, management, and digital sociology, with limited cross-sectoral synthesis.

Recent empirical research further indicates that workplace loneliness can diminish employees’ work engagement, which in turn increases job dissatisfaction; however, this relationship may be buffered in individuals with a stronger need to belong [1]. Therefore, understanding both the direct and indirect effects of workplace loneliness on subjective well-being, as well as the personal and organizational factors that mitigate its impact, remains an important research priority [43].

Evidence specifically addressing workplace loneliness in digital contexts is scarce, and available evidence varies regarding the role of digital tools such as videoconferencing, instant messaging platforms, or collaborative applications in fostering social connectedness. For some older workers, these technologies act as enablers of engagement, whereas for others—particularly those with lower digital literacy—they may inadvertently exacerbate feelings of exclusion and marginalization [31,32,53]. Contextual factors such as organizational culture, leadership style, and opportunities for informal interaction appear to significantly moderate these outcomes [30,33].

The paucity of targeted studies presents both a challenge and an opportunity. The challenge lies in the absence of comprehensive conceptual frameworks explicitly linking workplace loneliness to digital transformation among older professionals. The opportunity lies in the potential to design and evaluate targeted strategies—such as structured digital skill training, leadership-led initiatives to enhance social connection, and workplace policies promoting inclusive communication—that could strengthen older workers’ social integration and mitigate loneliness in increasingly digitalized work environments [54-56].

### Limitations

Our inclusion criteria restrict the review to studies providing empirical evidence published in English. This language limitation may introduce bias toward research conducted in anglophone or Western contexts and could affect the diversity of perspectives represented. Moreover, the existing body of literature directly addressing the intersection of workplace loneliness, digitalization, and older workers is notably limited. This scarcity presents both a challenge and an opportunity: while it constrains the available evidence for synthesis, it also underscores the novelty of the topic and the potential for this review to highlight significant gaps in current knowledge.

The term *workplace loneliness* was selected as the core concept for its alignment with our research objectives. The search strategy, developed in collaboration with an information specialist, incorporated both controlled vocabulary and free-text keywords to maximize coverage. Controlled terms ensure consistency in indexing, whereas free-text keywords capture variations in author language and disciplinary framing. Nonetheless, given the variability in terminology and the evolving nature of research in digitalized work environments, it remains possible that some relevant studies will not be retrieved despite these measures.

### Comparison With Previous Work

This scoping review protocol on workplace loneliness among older professionals (aged ≥50 years) in the context of digitalization uniquely addresses a gap not covered by previous literature syntheses. Previous reviews have largely examined loneliness and digital technologies among older adults in general without focusing specifically on those engaged in professional roles within digitally transforming workplace environments, such as the reviews on loneliness at work listed in Table 1. For example, Chen et al [50] investigated the effects of online social networking and information and communications technology tools on loneliness and mental health among older adults aged 50 to 83 years, but their work concentrated on community or home settings rather than workplace contexts. Similarly, Noone et al [48] assessed the impact of video calls on loneliness in adults aged ≥65 years residing in care homes, finding very low-certainty evidence and no applicability to professional environments. Hoang et al [49] provided a broader mapping of digital interventions—ranging from social media to virtual reality and assistive technologies—designed to reduce social isolation in older adults but excluded employment-related experiences.

**Table 1:** Selected systematic reviews on digital technologies for loneliness among older people at work.

Review	Population age (years)	Digital technologies	Type of loneliness
Noone et al [48], 2020	≥60	Video calls using Skype, FaceTime, and Zoom to connect with family members, friends, and health professionals	Loneliness (subjective): feelings of being alone or lacking companionship; social isolation (objective): lack of social contact or interaction
Chen et al [50], 2022	50-83	Online social networking sites such as Facebook and Twitter; communication tools: email, Skype, and instant messaging; information and communications technology generally, including forums and support groups; computer-mediated communication in some cases	Social isolation (objective): lack of social contacts or infrequent interaction; loneliness (subjective): perceived lack of meaningful connection; emotional loneliness (missing close or intimate relationships); social loneliness (lack of broader social network)
Hoang et al [49], 2022	≥60	Communication technologies: video calls, social media, and messaging platforms; social engagement platforms: online communities and forums; assistive and ambient technologies: robotic pets and virtual assistants (eg, Alexa)	Loneliness (subjective): feelings of being alone and disconnected; social isolation (objective): reduced or infrequent social contact

Few studies address the emotional and relational consequences of workplace digitalization—such as loss of informal contact, perceived isolation, or reduced social integration—in professionals aged 50 years or older. This clear research gap underscores the need for a focused synthesis that can clarify conceptual boundaries, identify sector-specific risk factors, and assess effective intervention strategies. This scoping review aims to provide such an integrated understanding and inform evidence-based approaches for fostering social connectedness among older professionals in increasingly digitalized work settings.

This review is distinguished by its emphasis on workplace digitalization, including remote work models, digital communication platforms, and automation, and its targeted focus on older workers still engaged in paid employment. Grounded in sociocognitive, life span developmental, and organizational theories, it conceptualizes loneliness as a context-dependent construct shaped by digital communication norms, leadership, and individual adaptation. Furthermore, the review aims to not only document digital tools associated with loneliness but also map interventions implemented by organizations to mitigate its effects. By focusing on professional inclusion and adaptation in the digital era, this review offers both theoretical and applied contributions to aging, organizational behavior, and digital workplace research.

### Conclusions

This scoping review will systematically investigate the ways in which digitalization influences the experience of workplace loneliness among professionals aged 50 years or older. By mapping the existing empirical and conceptual literature, the review will identify how digital tools, communication practices, and organizational changes contribute to shaping social connection—or the lack thereof—within professional settings. In doing so, it will provide a nuanced account of the interplay between technological transformation and the relational dynamics of later-stage careers.

Beyond documenting patterns in the evidence, the synthesis will also examine organizational strategies and interventions aimed at reducing workplace loneliness in digitally mediated environments. This dual focus will allow the review to refine conceptual clarity; uncover significant gaps in current knowledge; and offer insights that can guide the design of inclusive, socially supportive digital work environments. In turn, these findings have the potential to inform both policy development and practical initiatives that address the unique needs of an aging workforce.

## Appendix

Multimedia Appendix 1 PRISMA-ScR (Preferred Reporting Items for Systematic Reviews and Meta-Analyses extension for Scoping Reviews) checklist.

Multimedia Appendix 2 Anticipated PRISMA-ScR (Preferred Reporting Items for Systematic Reviews and Meta-Analyses extension for Scoping Reviews) flow diagram.

## Abbreviations

PCC: population, concept, and context
PRISMA-ScR: Preferred Reporting Items for Systematic Reviews and Meta-Analyses extension for Scoping Reviews
