# Supplementary material for: Workplace Loneliness Experience Among Older Professionals (Aged ≥50 Years) in the Context of Digitalization: Protocol for a Scoping Review
Source: JMIR Res Protoc. 2025 Dec 25;14:e81843. doi: 10.2196/81843 (PMC12784142; doi:10.2196/81843)
Supplement: Multimedia Appendix 1 [file resprot_v14i1e81843_app1.pdf]

Țânculescu-Popa, L., Brandão, M. P., Aksoy, Ș., Ilgaz, A., Hirata, R. P., Jakovljevic, M., & Tofan, C. (2025, May 21). *Exploring workplace loneliness and digitalization among older professionals (50+): Scoping review protocol*. OSF Registries. Protocol registered with the Open Science Framework. https://doi.org/10.17605/OSF.IO/6P4AK

Multimedia Appendix 1: PRISMA-ScR checklist

Table of contents

Study details according to the PRISMA-ScR checklist 2
Item 1. Title 2
Item 2. Abstract 2
Item 3. Rationale 2
Item 4. Objectives 3
Item 5. Protocol and registration 3
Item 6. Eligibility criteria 3
Item 7. Information sources 3
Item 8. Search 4
Item 9. Selection of sources of evidence 4
Item 10. Data charting process 4
Item 11. Data items 4
Item 12. Critical appraisal of individual sources of evidence 5
Item 13. Summary measures 5
Item 14. Synthesis of results 5
Item 15. Risk of bias across studies 5
Item 16. Additional analyses 5
Items 17-26: Results / Discussion 5
Item 27: Funding 6
References 6

Study details according to the PRISMA-ScR checklist

This study employs a scoping review design, adhering to the Preferred Reporting Items for Systematic Reviews and Meta-Analyses Extension for Scoping Reviews (PRISMA-ScR) checklist [1]. The current document is a short summary of the protocol that is currently undergoing peer-review in the journal JMIR Research Protocol.

Item 1. Title

Workplace Loneliness Experience Among Older Professionals (50+) in the Context of Digitalization: A Protocol for a Scoping Review.

Item 2. Abstract

Background: Workplace loneliness—defined as the perceived absence of meaningful social relationships at work— can have a negative impact on the well-being, engagement and productivity of employees. Older professionals (aged 50 and above) may be particularly vulnerable to workplace loneliness in the context of accelerated digitalization, which may create obstacles to inclusion, communication, and collaboration. Despite growing interest in this phenomenon, no comprehensive synthesis has yet examined how digital tools and transformations affect loneliness among older workers or what interventions have been implemented to address it.

Objective: This scoping review aims to systematically map the existing literature on workplace loneliness among workers aged 50 and over, with a particular focus on how digitalization influences these experiences. The review will also identify digital tools associated with loneliness and and explore organizational interventions to reduce loneliness among this demographic.

Methods: This scoping review will follow Arksey and O'Malley's (2005) methodological guidelines [1] and the PRISMA-ScR framework [2]. A comprehensive search will be conducted in multiple databases (e.g., MEDLINE, Scopus, Web of Science, APA PsycINFO, EMBASE, CINAHL, IEEE Xplore, and the ACM Digital Library) and grey literature. Eligible studies will (1) include workers aged 50+, (2) address workplace loneliness or related constructs in professional contexts, and (3) are situated in digitalized work environments (e.g., remote/hybrid work, digital tools, ICT systems). Study selection, data extraction, and quality assessment will be performed independently by multiple reviewers. Data will be charted using a predefined template covering study characteristics, theoretical framework, digital context, loneliness measures, and intervention strategies, and synthesized narratively and thematically.

Results: As this is a scoping review protocol, results are not yet available. A preliminary search conducted in June–July 2025 across MEDLINE, Cochrane Library, and Web of Science yielded 450, 1, and 47 potentially relevant records, respectively. No systematic or scoping reviews were identified on workplace loneliness among older workers in digitalized contexts. One review addressed video calls for non-working older adults, reporting very low-certainty evidence. The planned review will apply PRISMA-ScR guidelines to synthesize evidence on digitalization's role, associated technologies, and organizational interventions in mitigating workplace loneliness for professionals aged 50 and over.

Conclusions: This scoping review will systematically examine how digitalization shapes workplace loneliness among professionals aged 50 and over, and identify organizational interventions that address it. The synthesis will refine conceptual understanding, highlight critical evidence gaps, and inform the development of socially supportive digital work environments for aging workforces.

Item 3. Rationale

This scoping review aims to identify and synthesize existing evidence on how digitalization influences the perceived workplace loneliness experienced by older workers

Item 4. Objectives

The objective of this scoping review is to identify the scientific literature addressing workplace loneliness among older workers (aged 50+), with a particular focus on the role of digitalization. Accordingly, the review will examine the following topics:

1. Study design and primary focus.
2. Type of digitalization or digital tools involved.
3. Employment setting and organizational context.
4. Reported effects on workplace loneliness and related interventions.
5. Evidence gaps in this field.

Item 5. Protocol and registration

The study was registered at the Open Science Framework (https://osf.io/6p4ak)

Item 6. Eligibility criteria

The eligibility criteria for this scoping review are based on the Population, Concept and Context (PCC) criteria:

Inclusion criteria

1. Population: professionals aged 50 years and older, or studies with age-disaggregated data allowing analysis for this group.
2. Concept: workplace loneliness, professional isolation, solitude, or emotional disconnection in professional settings.
3. Context: digitalized work environments (e.g., remote or hybrid work, digital communication platforms, automation, digital transformation).
4. Setting: professional or organizational contexts involving paid employment.
5. Study type: original studies with any design or data type (quantitative, qualitative, or mixed-methods), including dissertations and organizational reports with empirical data.
6. Publication status: peer-reviewed journal articles, conference proceedings, dissertations, and reputable grey literature (e.g. reports from reputable organizations)
7. Publication language: English
8. Time frame: published from 2000 onwards.
9. Full-text availability: full-text accessible for review.

Exclusion criteria:

1. Population: studies focusing exclusively on younger workers (<50) or on retirees not engaged in professional work.
2. Concept: general or personal loneliness unrelated to the workplace, or studies on workplace factors without specific reference to loneliness or social disconnection.
3. Context: non-digitalized work environments or settings without reference to technology-mediated changes in the workplace.
4. Setting: informal, non-professional, or volunteer work contexts.
5. Study type: protocols, narrative reviews, systematic reviews, opinion pieces, editorials, or non-empirical publications.
6. Publication status: unpublished studies lacking peer review, low-quality grey literature without empirical data.
7. Publication language: languages other than English
8. Time frame: published before 2000.
9. Full-text availability: full-text not accessible for review.

Item 7. Information sources

The information sources for this scoping review will include the following international bibliographic databases: MEDLINE (via PubMed), Cochrane Library, ProQuest (including Dissertations & Theses Global), Web of Science Core Collection, Scopus, APA PsycINFO, ERIH PLUS, EMBASE, CINAHL, IEEE Xplore, and the ACM Digital Library. These sources will be used to identify relevant scientific studies in accordance with the PCC-defined eligibility criteria.

Item 8. Search

The electronic search strategy was developed and tested by the authors, using Boolean operators (AND, OR) and truncation symbols to capture relevant terms related to loneliness, digitalization, and older workers. The final search strategy will be adapted for each database and fully reported in the scoping review.

Item 9. Selection of sources of evidence

References will be imported into COVIDENCE, where duplicates will be removed before proceeding with title and abstract screening, followed by full-text review. A standardized data extraction template will capture key details, including participant demographics, employment setting, type of digitalization, workplace loneliness outcomes, and organizational interventions. Each record will be independently reviewed by two authors, with disagreements resolved through discussion or a third reviewer. Regular team meetings will ensure consistent application of eligibility criteria.

Item 10. Data charting process

The initial data charting form was developed and refined by the protocol authors to ensure alignment with the research objectives (see Item 11 for proposed items). Following Levac et al. [3], data extraction will be treated as an iterative process, allowing for revisions to the form as familiarity with the evidence base increases. Additional items may be incorporated if deemed relevant for answering the research questions. Consistent with the recommendations of Daudt et al. [4], two researchers will pilot the form on a sample of seven articles to evaluate its clarity and completeness. The form will be reviewed and finalized by the research team before use. Data extraction will then be performed independently by two reviewers in an Excel spreadsheet, with discrepancies resolved through discussion or, if necessary, by a third team member.

Item 11. Data items

Data corresponding to the items listed below, along with their descriptions, will be extracted from the articles to address the objectives of this scoping review. For internal purposes, the reviewer's name will be recorded at the top of the form.

**Item**	**Description**
Author(s), Year (APA style)	If 1 author: Author Surname (year). If 2 authors: Author and Author (year). If 3+ authors: First author et al. (year)
Title of the Study	Full title of the article
N (number of participants)	
N in follow-ups	For example, in intervention or randomized control studies: How many participants responded in a follow-up (e.g., after 1 year)?
Country/Region	Country or geographical context where the study was conducted
Type of Publication	Article type: empirical study, conceptual paper, review, thesis, etc.
Theoretical Framework	Any theories or conceptual models used
Aim / Objective of Study	Main research aim or objective stated by the authors
Study design	Methodology used: quantitative, qualitative, mixed methods, case study, longitudinal, etc.
Sample Characteristics	Description of participants: age (especially 50+), job roles, industry, sample size, gender, etc.
Type of population	e.g. employees, employers, general population
Type of workers	Occupation/Employment branch

Study includes both young and older workers?	Studies with young people may only be included if older workers are also represented and analyzed (aged 50 years and above)
Includes both older adults and older workers?	Only studies focusing on older workers (aged 50 years and above) will be included; studies addressing older adults who are not engaged in paid employment will be excluded.
Gender/sex used in analysis/results	When participant data are reported separately by gender, mark the corresponding boxes (“female”, “male”). When data are presented for the entire sample without gender breakdown, mark “total f+m”.
Digitalization context	How digitalization is defined or contextualized (e.g., remote work, communication platforms, automation)
Digital Tools / Technologies	Specific digital tools/platforms mentioned (e.g., Zoom, MS Teams, email systems, digital dashboards)
Operational Definition of Loneliness	How loneliness is defined in the study (e.g., emotional loneliness, social isolation, workplace alienation)
Measures / Indicators of Loneliness	Scales or instruments used to measure loneliness (e.g., UCLA Loneliness Scale, interviews, etc.)
Key Findings on Digitalization and Loneliness	Main findings about how digitalization affects loneliness and whether digital tools are directly associated with loneliness (positively or negatively), or act as mediators/facilitators
Moderators / Mediators	Any variables that moderate or mediate the relationship (e.g., digital literacy, leadership, age)
Positive / Negative Effects	Discussion of both beneficial and harmful effects of loneliness (e.g., solitude vs. isolation)
Type of Intervention	Any intervention implemented (e.g., digital training, mentorship programs, social inclusion strategies)
Intervention Strategy	How the intervention was implemented: tools, duration, content topics
Intervention Content	Titles and brief summaries of the intervention content
Intervention Outcomes	Outcomes or results of the intervention (e.g., reduced loneliness, increased digital competence)
Comments / Notes	Additional notes, observations, or key quotes

Item 12. Critical appraisal of individual sources of evidence

Although critical appraisal is not typically required in scoping reviews [1], the methodological quality of included studies will be assessed using the Joanna Briggs Institute (JBI) Critical Appraisal Checklists appropriate to each study design [5,6]. Discrepancies will be resolved by consensus.

Item 13. Summary measures

Not applicable for scoping reviews.

Item 14. Synthesis of results

The extracted data will be organized in table format and presented through a narrative synthesis.

Item 15. Risk of bias across studies

Not applicable for scoping reviews.

Item 16. Additional analyses

Not applicable for scoping reviews.

Items 17-26: Results / Discussion

Not applicable at the protocol stage.

Item 27: Funding

The research was partially financed through the COST Action CA21107 “Work inequalities in later life redefined by digitalization” (DIGI-net) that is supported by the European Cooperation in Science and Technology (COST).(https://www.cost.eu/actions/CA21107/).

References

1. Arksey, H., & O'Malley, L. (2005). Scoping studies: towards a methodological framework. *International journal of social research methodology*, *8*(1), 19-32.
2. Tricco, A. C., Lillie, E., Zarin, W., O'Brien, K. K., Colquhoun, H., Levac, D., ... & Straus, S. E. (2018). PRISMA extension for scoping reviews (PRISMA-ScR): checklist and explanation. *Annals of internal medicine*, *169*(7), 467-473.
3. Levac, D., Colquhoun, H., & O'Brien, K. K. (2010). Scoping studies: advancing the methodology. *Implementation science*, *5*(1), 69.
4. Daudt, H. M., Van Mossel, C., & Scott, S. J. (2013). Enhancing the scoping study methodology: a large, inter-professional team’s experience with Arksey and O’Malley’s framework. *BMC medical research methodology*, *13*(1), 48.
5. Liberali, R., Kupek, E., & Assis, M. A. A. D. (2020). Dietary patterns and childhood obesity risk: a systematic review. Childhood obesity, 16(2), 70-85.
6. Peters, M. D., Marnie, C., Tricco, A. C., Pollock, D., Munn, Z., Alexander, L., ... & Khalil, H. (2020). Updated methodological guidance for the conduct of scoping reviews. *JBI evidence synthesis*, *18*(10), 2119-2126.
