# Supplementary material for: Workplace Loneliness Experience Among Older Professionals (Aged ≥50 Years) in the Context of Digitalization: Protocol for a Scoping Review
Source: JMIR Res Protoc. 2025 Dec 25;14:e81843. doi: 10.2196/81843 (PMC12784142; doi:10.2196/81843)
Supplement: Multimedia Appendix 2 [file resprot_v14i1e81843_app2.pdf]

Țânculescu-Popa, L., Brandão, M. P., Aksoy, Ş., Ilgaz, A., Hirata, R. P., Jakovljevic, M., & Tofan, C. (2025, May 21). *Exploring workplace loneliness and digitalization among older professionals (50+): Scoping review protocol*. OSF Registries. Protocol registered with the Open Science Framework. https://doi.org/10.17605/OSF.IO/6P4AK

**Multimedia Appendix 2: Anticipated PRISMA-ScR flow diagram.**

The figure illustrates the planned study selection process, following PRISMA-ScR guidelines (Tricco et al., 2018).

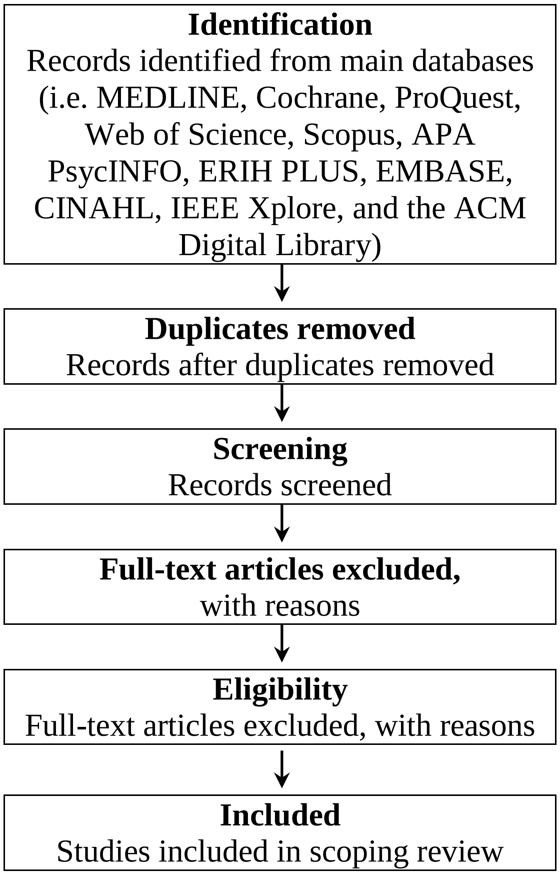


**Figure 1:** Anticipated PRISMA-sCR flow diagram illustrating the planned study selection process.

*Note*: Numerical data will be populated upon completion of the review.
